# De novo full length transcriptome analysis and gene expression profiling to identify genes involved in phenylethanol glycosides biosynthesis in *Cistanche tubulosa*

**DOI:** 10.1186/s12864-022-08921-x

**Published:** 2022-10-08

**Authors:** Lei Hou, Guanghui Li, Qingliang Chen, JinJin Zhao, Jiaowen Pan, Ruxia Lin, Xiujin Zhu, Pengfei Wang, Xingjun Wang

**Affiliations:** 1 Institute of Crop Germplasm Resources, Shandong Academy of Agricultural Sciences, Shandong Provincial Key Laboratory of Crop Genetic Improvement, Ecology and Physiology, Jinan, 250100 China; 2 Modern Research Center for Traditional Chinese Medicine, Beijing University of Chinese Medicine, Beijing, 100029 China; 3 Shandong Academy of Grape, Shandong Engineering Research Center for Grape Cultivation and Deep-Processing, Jinan, 250100 China; 4 College of Agronomy, Hebei Agricultural University, Baoding, 071000 China

**Keywords:** *Cistanche tubulosa*, Full-length transcriptome, PacBio; RNA-seq, Phenylethanol glycosides

## Abstract

**Background:**

The dried stem of *Cistanche,* is a famous Chinese traditional medicine. The main active pharmacodynamic components are phenylethanol glycosides (PhGs). *Cistanche tubulosa* produces higher level of PhGs in its stems than that of *Cistanche deserticola*. However, the key genes in the PhGs biosynthesis pathway is not clear in *C. tubulosa*.

**Results:**

In this study, we performed the full-length transcriptome sequencing and gene expression profiling of *C. tubulosa* using PacBio combined with BGISEQ-500 RNA-seq technology. Totally, 237,772 unique transcripts were obtained, ranging from 199 bp to 31,857 bp. Among the unique transcripts, 188,135 (79.12%) transcripts were annotated. Interestingly, 1080 transcripts were annotated as 22 enzymes related to PhGs biosynthesis. We measured the content of echinacoside, acteoside and total PhGs at two development stages, and found that the content of PhGs was 46.74% of dry matter in young fleshy stem (YS1) and then decreased to 31.22% at the harvest stage (HS2). To compare with YS1, 13,631 genes were up-regulated, and 15,521 genes were down regulated in HS2. Many differentially expressed genes (DEGs) were identified to be involved in phenylpropanoid biosynthesis pathway, phenylalanine metabolism pathway, and tyrosine metabolism pathway.

**Conclusions:**

This is the first report of transcriptome study of *C. tubulosa* which provided the foundation for understanding of PhGs biosynthesis. Based on these results, we proposed a potential model for PhGs biosynthesis in *C. tubulosa*.

**Supplementary Information:**

The online version contains supplementary material available at 10.1186/s12864-022-08921-x.

## Introduction

*Cistanche tubulosa* (Schenk) R. Wight, a valuable Chinese herb medicine*,* is a perennial parasitic plant specifically parasitic on the roots of *Tamarix ramosissima*. Besides growing in the desert area, *T. ramosissima* is also widely distributed in the saline-alkali area in eastern China, which provides the opportunity for planting *C. tubulosa* in saline-alkali land. In recent years, *C. tubulosa* has been successfully introduced into the saline areas of the Yellow River Delta in China [1]. Growing *C. tubulosa* either in the desert area or in saline-alkali land could bring great values both economically and ecologically.

Phenylethanol glycosides (PhGs), oligosaccharides, and iridoids are the main active pharmacodynamic components in *C. tubulosa*. PhGs, in particular, draw the most attention of the researchers because of their promising pharmacological characteristics. Studies have shown that PhGs has outstanding biological activities such as kidney tonifying, bloodand essence invigorating, intestines moistening and defecation, anti-aging, learning ability improvement, nerve and liver protection [2–6]. *C. tubulosa* accumulates more PhGs than *C. deserticola*, in particular echinacoside and verbascoside, which is also the more suitable species for cultivation due to its high yield [7, 8]. The biosynthesis of PhGs is highly regulated by both environment stimuli and development progress. The processing condition after harvest also significantly affect the content of PhGs. The difference in PhGs contents of different samples are significant, even for the same species. Previous studies indicated that the total contents of seven PhGs components of *C. tubulosa* from south of Xinjiang were approximately six times those of Kuitun and Hami in China [9]. The accumulation of PhGs varies in different part of the fresh succulent stem or different growth stages of *C. tubulosa*, and the young stem accumulate more PhGs than that of the old stem [10–12]. The content of active components in different parts of *C. tubulosa* was also different in different development stages. The content of PhGs in the base part of *C. tubulosa* fleshy stem was the highest, and in the top part was the lowest. The echinoside content of the base part was 2 ~ 8 times as higher as the top part [13, 14].

Three major components of PhGs are organic acid, saccharide, and phenylethanol aglycon [17]. Referring to the studies of *Rehmannia glutinosa* and *C. deserticola,* PhGs biosynthetic pathway is related to phenylpropanoid biosynthesis pathway (PS, Ko00940), phenylalanine metabolism pathway (PM, Ko00360), and tyrosine metabolism pathway (TM, Ko00350) [17]. However, the regulation of PhGs biosynthetic pathway and key enzymes in *C. tubulosa* is unclear. To uncover the biosynthesis and regulation of PhGs, to stabilize and improve the content of PhGs are all important research areas in *Cistanche*. However, little molecular information is available in *C. deserticola* except few studies on chloroplast genome sequencing and transcriptome analysis [15–17]. There is even less genomic and molecular information available in *C. tubulosa.* The limited genomic and molecular information of this species hinders the understanding of the molecular mechanisms of PhGs biosynthesis and regulation. The third-generation single-molecule real-time sequencing platform (SMRT) can capture the full-length transcripts and avoid the assembly process [18]. SMRT sequencing has been successfully used in many plant species for high quality full-length transcripts identification and transcriptome analysis [19–22]. Full-length transcriptome analysis is a powerful way for revealing the dynamics of gene expression, and understanding the molecular mechanism for complex biological processes.

In this study, the PacBio SMRT Sequel platform was employed to generate a full-length transcriptome of *C. tubulosa*. In addition, RNA-seq technology was used to investigate the gene expression dynamics of C. *tubulosa* in different developmental stages. Our study generated a high-quality transcriptome reference sequence of C. *tubulosa*, and identified the key metabolic enzyme genes for PhGs biosynthesis. These results lay the foundation for understanding the molecular mechanism for PhGs biosynthesis.

## Materials and methods

### Plant materials and RNA sample preparation

The fleshy stems and flower organs of *C. tubulosa* at different developmental stages were collected from an experimental field of Shandong Academy of Agricultural Sciences in Weifang City, China. Four materials were collected for full-length sequencing with PacBio, including young succulent stem, succulent stem at harvest stage, succulent stem at flowering stage, and the inflorescence part above ground. After cleaning, the tissues were cut into small pieces and immediately frozen in liquid nitrogen, and stored at -80 °C until further processing. The total RNA samples were isolated using RNeasy Plus Mini Kit (Qiagen) according to the protocol of manufacturer (http://www.qiagen.com). The quantity and quality of total RNA was assessed using Agilent 2100 Bioanalyzer and Fragment Analyzer Automated CE System (http://www.agilent.com). For each sample, three biological replications were prepared.

### Library preparation and high-throughput sequencing

The PacBio UMI Iso-Seq sequencing library was constructed using equally mixed RNAs from different tissues. First-strand cDNA was synthesized using UMI base PCR cDNA Synthesis Kit (BGI). After synthesis of first strand, PCR amplification was performed to generate double-strand cDNA. Then, multiple transcripts are connected end to end to generate multifold flux full-length transcriptome Sequel PacBio IsoSeq library. The problem of different size fragment preference was solved by Sequel platform. The library was subsequently sequenced using a PacBio Sequel system.

### PacBio Iso-Seq data processing and bioinformatics analysis

After sequenced by PacBio sequel, large number of Circular Consensus Sequencing (CCS) reads were obtained. Reads of insert (ROI) was identified and classified into full-length (FL) and non-full-length (non-FL) reads. Then the high-quality full-length consistent sequence were obtained and evaluated [23]. The high-quality full-length sequences of two libraries were combined for clustering to de-redundancy and isoform expression quantification. The TransDecoder (https://transdecoder.github.io) was first used for recognizing the longest Open Reading Frames (ORFs), then the ORFs were further blasted with SwissProt (http://ftp.ebi.ac.uk/pub/databases/swissprot) and Pfam (http://pfam.xfam.org). Five other public databases were used to annotate the transcripts, including Nt and Nr of NCBI (http://www.ncbi.nlm.nih.gov/), GO (http://geneontology.org), KOG (http://www.ncbi.nlm.nih.gov/KOG) and KEGG ([24, 25]. HMMseach software (http://hmmer.org) was used for searching the plant transcription factors database (http://plntfdb.bio.uni- potsdam.de/v3.0/). CPC, txCdsPredict, and CNCI softwares and Pfam database were used to predict the coding and non-coding sequences.

### Bioinformatics Analysis of RNA-Seq Data

For RNA-seq, the young fleshy stem (YS1) and the harvest stage stem (HS2) samples were used for gene expression analysis. Libraries were constructed using the method described in previous studies [26]. Briefly, the mRNA was first enriched and cleaved into short fragments, and then used to synthesize cDNAs. The cDNA fragments were purified and enriched through PCR amplification to construct cDNA library. Sequencing was conducted using BGISEQ-500 platform (BGI, China). The clean reads were obtained by removing the reads with poly *N* > 10%, reads containing adaptor sequences, and low-quality reads through SOAPnuke (version 1.5.2) [27]. Then, all clean reads were mapped with the full-transcripts of *C. tubulosa* using Bowtie2 (version 2.2.5) software [28]. The gene expression level was calculated using RSEM, and normalized using FPKM (Fragments Per Kilobase of transcript per Million fragments mapped) method [29]. The relative gene expression level between different samples was calculated using R package DEGseq [30]. Differentially expressed genes (DEGs) were identified following the criteria of fold change >  = 2 and Q-value <  = 0.05. To further understand the function of the DEGs, GO analysis was performed and enriched GO terms were identified using Blast2GO using hypergeometric test comparing with the whole transcript background [26, 31]. In addition, enriched KEGG pathways were identified by comparing the ratio of DEGs with the whole transcript background.

### Verification of RNA-seq data by qRT-PCR

qRT-PCR was used to verify the expressionlevels of 10 selected genes. RNA samples were those used for high-throughput sequencing and the reverse transcription was performed using PrimeScript II 1st Strand cDNA Synthesis Kit (TaKaRa). The gene-specific primers were designed using PerlPrimer software and were listed in Additional Table S7. qRT-PCR reaction was performed on ABI7500 Real Time System (Applied Biosystems) using TB Green™ Premix Ex Taq™ II (TaKaRa). The parameters of thermal cycle were 94℃ for 10 min, followed by 30 cycles of 94℃ for 15 s and 60c for 1 min in a 20 µl volume. Three biological replications were performed for each reaction with actin gene as internal reference. The relative expression level of each gene between YS1 and HS2 was calculated by ^2−△△^Ct method.

### Preparation and quantification of PhGs

The freshly harvested *C. tubulosa* was washed and sliced into thin sections, then steamed at 100℃ temperature for 1 min, dried in the oven, and then ground into powder. To extract PhGs, 50 mL of 50% methanol was added into 1.0 g of *C. tubulosa* powder, and then ultrasonic treatment was conducted for 30 min (500 W, 40 kHz). Add 50% methanol to supplement the weight loss, shake well, stand, and collect the supernatant. The supernatant was filtered through 0.22 μm microporous membrane to obtain the phenylethanol glycosides extracts.

The contents of echinacoside and acteoside were determined by HPLC using a column of Phenomenex Luna 5 μm C18(A) column (4.6 × 250 mm, 5 μm). The isocratic mobile phase consisted of mobile phase A (methanol/acetonitrile, 1:6, v/v), and mobile phase B (0.1% formic acid). The gradient program is as followed: 90% ~ 82% B at 0 ~ 8 min; 82% ~ 76% B at 8 ~ 15 min; 76% ~ 74% B at 15 ~ 20 min; 74% ~ 70% B at 20 ~ 28 min; 70% ~ 65% B at 28 ~ 35 min. The total elution time was 35 min, with the flow rate of 1 ml/min. Column temperature was kept constant at 30 °C. Ultraviolet detection wavelength was 330 nm.

The contents of total PhGs were quantitated by ultraviolet spectrophotometry. Weigh 10 mg of echinosiden and put it in a 50 ml volumetric flask and dissolved in 50% methanol to obtain the standard solution. 0.2 mL, 0.4 ml, 0.6 ml, 0.8 ml, and 1.0 ml of standard solution were accurately aliquot to a 10 ml volumetric flask, diluted with 50% methanol to a final volume of 10 ml, and shaken well. The absorbance was measured at 330 nm to make the standard curve. The absorbance of the sample was measured at 330 nm, and the content of total PhGs was calculated according to the standard curve.

## Results

### Full-length transcriptome sequencing and de novo assembly

**Fig. 1:**
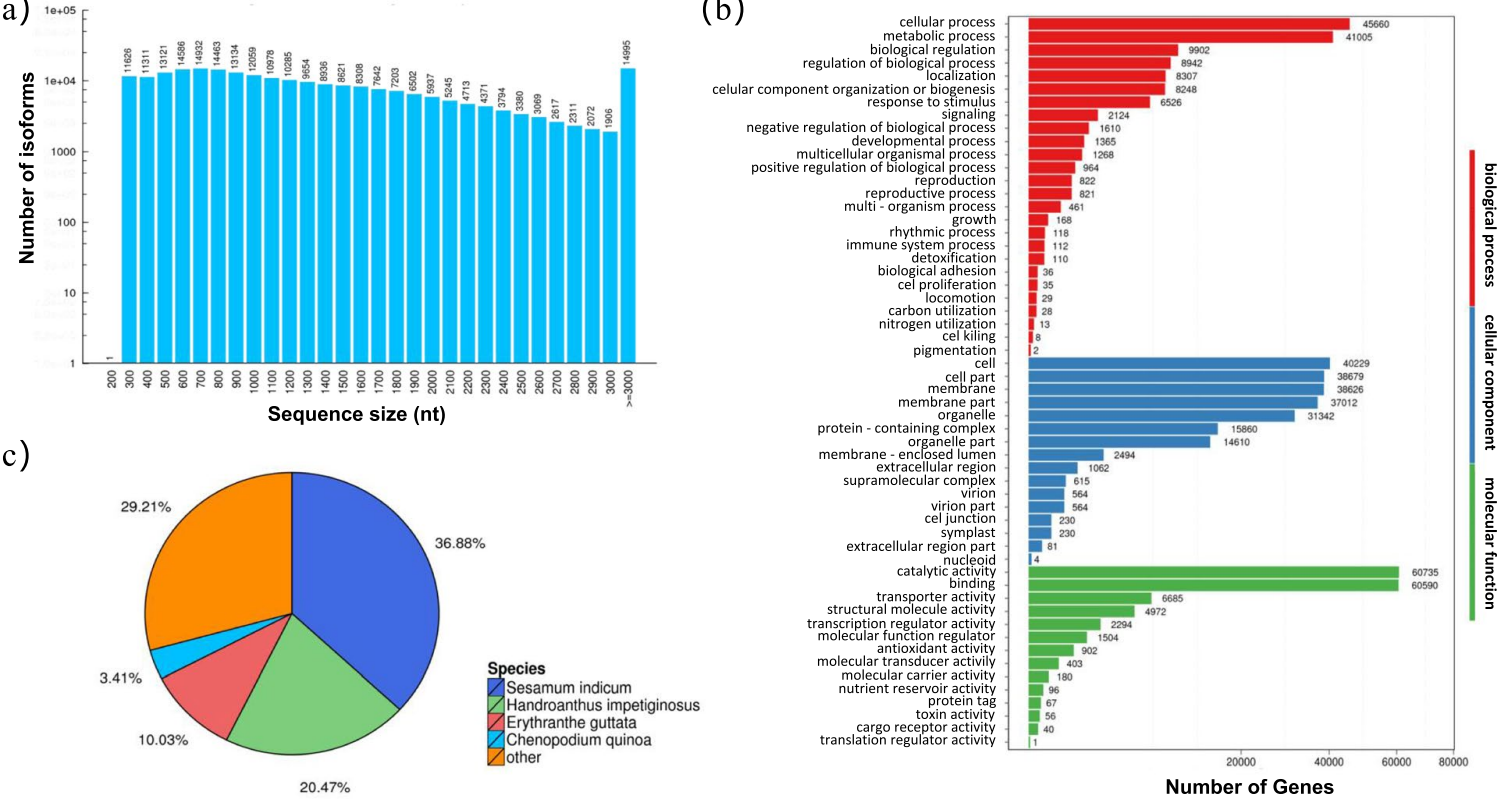
Characteristics of unique transcript isoforms of *C. tubulosa*. **a** Length distribution of the transcripts, **b** GO annotation of the transcripts, **c** Species mapped with the transcripts

Two PacBio IsoSeq libraries (F01, H01) were constructed by combining equal amount of total RNAs from four types of tissues including young succulent stem, succulent stem in harvest stage, succulent stem at flowering stage and the inflorescence above ground. PacBio sequencing generated a total of 21.87 Gb dataset including 4,321,117 sub-reads. A total of 254,813 Circular Consensus Sequencing (CCSs) reads were generated with the mean read length of 5,348 bp and 6,485 bp in the two libraries. SMRT analysis identified a total of 871,064 reads of full length non-chimeric transcripts (FLs) from CCSs. After removing the redundant sequences, a total of 237,772 unique isoforms (transcripts) were obtained. The transcript lengths were ranged from 199 bp to 31,857 bp, with a N50 of 1,797 bp. The GC content is 41.90% (Fig. 1a; Supplementary Table S1). BUSCO assessment showed that the transcripts are with high quality (Supplementary Figure S1).

To further test the integrity of the protein coding transcripts, the TransDecoder software was used for identifying the candidate coding regions in isoforms, through further blasted with the SwissProt and Pfam Database. In total, 127,241 transcripts (53.51%) showed significant homology to known proteins and contained the complete coding sequence (CDS). The PacBio sequencing provided large number of full-length transcripts of *C. tubulosa,* an important genomic resource for herb *Cistanche* research community*.*

### Functional annotation of the transcripts

To acquire the comprehensive annotation for these transcripts, the sequences were used for aligning with seven functional databases. The results showed that 174,391 (73.34%), 142,528 (59.94%), 122,202 (51.39%), 132,164 (55.58%), 130,770 (55.00%), 105,386 (44.32%), and 125,067 (52.60%) transcripts could match the Nr, Nt, SwissProt, KEGG, KOG, Pfam and GO databases, respectively (Supplementary Table S1). In total, there were 188,135 (79.12%) transcripts were annotated by more than one public databases. There were 29,622 transcripts were identified actively expressed with FPKM more than 0.5.

GO analysis grouped these genes into three categories: biological process, cell composition and molecular function. In terms of molecular function, catalytic activity and binding were the most abundant classes; in terms of cell, cell part, membrane and membrane part and organelle were the main classes; in terms of biological process, cellular process, and metabolic process were the most abundant classes (Fig. 1b). Nr annotation showed that 64,315 (36.88%), 35,698 (20.47%), and 17,491 (10.03%) transcripts matched the species of *Sesamum indicum*, *Handroanthus impetiginosus,* and *Erythranthe guttata*, respectively. The other 5,947 (3.41%) transcripts were matched with *Chenopodium quinoa* and the rest of 50,940 (29.21%) transcripts had similarity with other plant species (Fig. 1c).

**Fig. 2:**
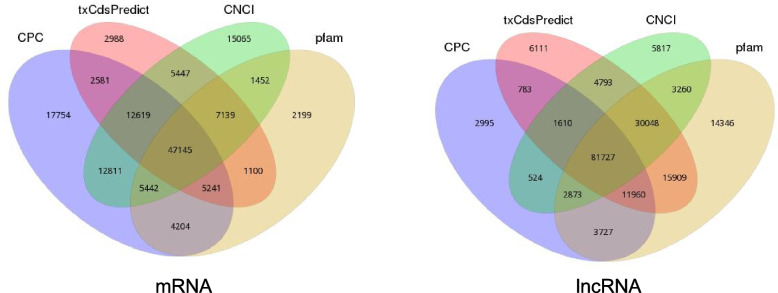
Venn diagram showed the prediction results of coding ability. Different colors represent different prediction methods

We found that the coding mRNA and the non-coding lncRNA accounted for 7,587(32.63%), and 128,218 (53.92%), respectively.(Fig. 2; Supplementary Table S2). In this study, a total of 3,862 (1.62%) transcripts encoding 57 types of TFs were identified through blasting with PlnTFDB database (Supplementary Table S3). Among these TFs, MYB, C3H, bHLH, AP2-EREBP, WRKY, NAC, and mTERF accounted for 50% of the total TFs, representing the most abundant TF families. The annotation of these transcripts provides a framework for future gene identification and gene transcriptionregulation in *C. tubulosa.*

### Determination of PhGs content in succulent stem

**Fig. 3:**
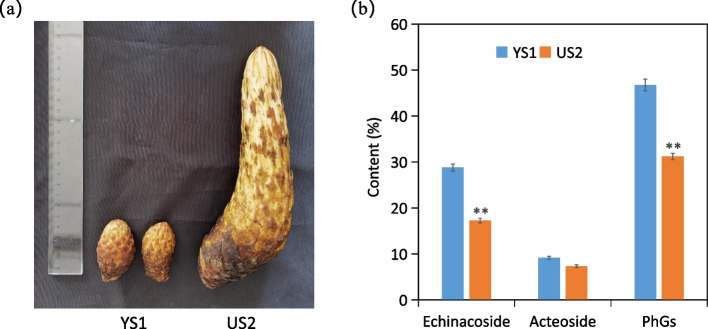
*C. tubulosa* samples and phenylethanol glycosides content of stem at different developmental stages. **a** YS1 and HS2 stem of *C. tubulosa*, **b** The contents of echinacoside, acteoside and total PhGs

PhGs, for example, acteoside, echinoside, salidroside, and isoacteoside are known to be the primary active molecules in *Cistanche*. We measured the content of echinacoside, acteoside, and total PhGs in YS1 and HS2 (Fig. 3). The results showed that PhGs content was 46.74% of dry matter in YS1, while it was 31.22% in HS2. The content of echinoside and acteoside was 28.82% and 9.17% in YS1, while 17.26% and 7.33% in HS2, respectively.

### Identification of enzyme genes in PhGs biosynthesis

PhGs biosynthetic pathway is related to PS, PM, and TM pathways, We analyzed the genes involved in these three pathways. Using the annotated full-length transcriptome data, we identified 1080 transcripts which encod 22 enzymes involved in PhGs biosynthesis (Table 1; Supplementary Table S4). In most cases, more than one unigenes were annotated as the same enzyme, and the transcripts number encoding UDP-glycosyltransferase were the most (185 transcripts) and followed by peroxidase (138 transcripts). Among these genes, the average length of copper amine oxidase, phenylalanine ammonia-lyase, and cinnamyl alcohol dehydrogenase transcripts were 2212.89 bp, 1977.84 bp, and 955.84 bp, respectively.

**Table 1:** Identified genes involved in phenylethanoid glycosides biosynthesis

Gene Name	Number of transcript	Average length / bp	Range of length / bp	Enzyme	Enzyme code	Pathway
*PAL*	42	1977.14	420–2891	Phenylalanine ammonia-lyase	4.3.1.24	PS
*C4H*	26	1486.88	536–5111	Cinnamic acid 4-hydroxylase/ trans-cinnamate 4-monooxygenase	1.14.14.91	PS
*C3H*	43	1649.42	716–2641	Coumarate 3-hydroxylase	1.14.13.-	PS
*CSE*	72	1159.69	215–2351	Caffeoylshikimate esterase	3.1.1.-	PS
*CYP98A3*	4	1904.25	1741–2168	5-O-(4-coumaroyl)-D-quinate 3’-monooxygenase	1.14.14.96	PS
*HCT*	13	1504.62	991–1804	Shikimate O-hydroxycinnamoyltransferase	2.3.1.133	PS
*4CL*	50	1660.84	334–3483	4-coumarate-CoA ligase	6.2.1.12	PS
*COMT*	80	1348.14	362–7048	Caffeic acid 3-O-methyltransferase	2.1.1.68	PS
*CCoAOMT*	17	1104.41	637–1873	Caffeoyl-CoA O-methyltransferase	2.1.1.104	PS
*F5H*	8	1318.25	488–2265	Ferulate-5-hydroxylase/Cytochrome P450 84A1	1.14.14.B13	PS
*PER*	138	1371.30	237–4360	Peroxidase	1.11.1.7	PS
*CCR*	51	1268.33	205–2230	Cinnamoyl-CoA reductase	1.2.1.44	PS
*CAD*	43	955.84	606–1909	Cinnamyl alcohol dehydrogenase	1.1.1.195	PS
*AADC*	13	*1366.85*	*440–1860*	Aromatic-L-amino-acid/L-histidine decarboxylase	4.1.1.28	PM; TM
*TyDC/DDC*	5	1437.88	440–1921	Tyrosine/DOPA decarboxylase	4.1.1.25	TM
*GOT*	42	1642.64	407–3452	Aspartate aminotransferase	2.6.1.1	TM
*hisC*	6	*1349.56*	*702–3738*	Histidinol-phosphate aminotransferase	2.6.1.9	TM
*TAT*	2	*1629.5*	*1626–1633*	Tyrosine aminotransferase	2.6.1.5	TM
*CuAO*	61	2212.89	775–3596	Copper amine oxidase	1.4.3.21	PM; TM
*PPO*	62	1597.3	292–2771	Polyphenol oxidase/Catechol oxidase	1.10.3.1	TM
AADH	117	1214.95	344–2192	aryl-alcohol dehydrogenase/alcohol dehydrogenase	1.1.1.90	PM; TM
UGT	185	1470.6	289–7842	UDP-glycosyltransferase/UDP-glucosyl transferase	2.4.1.-	-

### Differentially expressed genes (DEGs) between different developmental stages

RNA-seq was performed to quantify the transcript abundance in YS1 and HS2. Six samples were sequenced using DNBSEQ-500 platform, and on average 21.59 Mb clean reads were generated from each sample. About 87.87% of the reads were mapped to the full-length transcriptome. A total of 175,162 unigenes were identified expressing in these two stages. We used a stringent cutoff of FPKM ≥ 1 to define transcripts that were robustly expressed in specific tissue. Among these transcripts, 75,719 (43.23%) in YS1 and 75,214 (42.94%) in HS2 were detected.

**Fig. 4:**
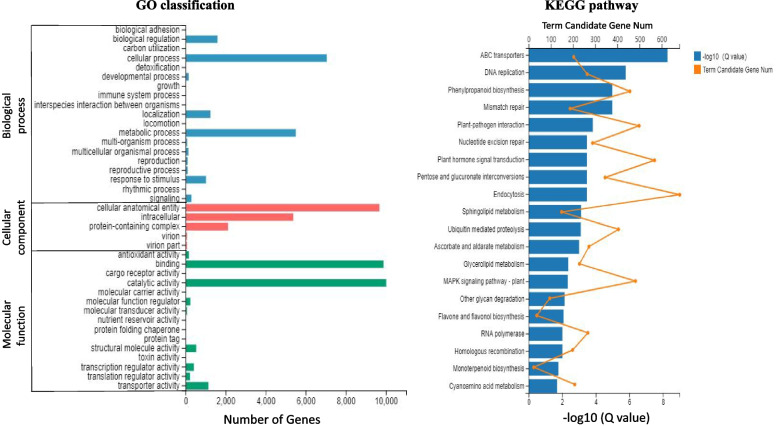
GO classification and KEGG pathway enrichment analysis

Between these two stages, 29,152 DEGs were identified (Supplementary Table S5). To compare with YS1, there were 13,631 and 15,521 up- and down-regulated genes in HS2. The DEGs were classified into three main GO categories: biological process, cellular component, and molecular function (Fig. 4). Cellular process and metabolic process were the top two terms in the biological process. In cellular component category, DEGs were mainly distributed in terms of cellular anatomical entity and intracellular. The most abundant terms in the molecular function were catalytic activity and binding. KEGG analysis classified the DEGs into 136 metabolic pathways (Supplementary Table S6). ABC transporters, DNA replication, phenylpropanoid biosynthesis, plant-pathogen interaction, plant hormone signal transduction were observed in the top 20 enriched pathways (Fig. 4). In addition, phenylalanine, tyrosine, and tryptophan biosynthesis pathway appeared in the top 30 enriched pathways, which were responsible for generating phenylethanol glycosides.

**Fig. 5:**
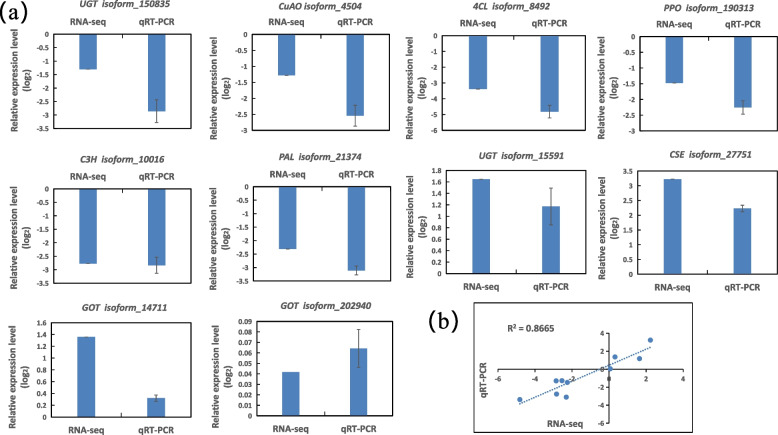
Verification of DEGs by qRT-PCR. **a** Transcript levels of 10 genes related to PhGs synthesis. Data are means of three replicates, and error bars represent ± SE (*n* = 3). **b** Pearson’s correlation of gene expression ratios between RNA-seq and qRT-PCR. The correlation of the fold change was analyzed by RNA-seq (x-axis) with qRT-PCR (y-axis) data

To further validate the RNA-seq results, 10 genes (four involved in PS pathway, three in TM pathway, two *UGT* genes and one *GuAO* gene) with different expression levels and functions were selected for qRT-PCR analysis. The qRT-PCR expressions of these genes were in accordance with the RNA-Seq results, with the correlation coefficient of 0.8665 (Fig. 5 and Supplementary Table S7).

### DEGs encoding transcription factors

Previous studies have shown that WARK, MYB, and bHLH transcription factors play key roles in regulation of phenylpropane synthesis pathways [32–34]. In our results, 27 Gene encoding *WARK* transcription factor were downregulated while only 6 genes were upregulated at HS2 stage. Most of genes that encode MYB and bHLH transcription factors were down-regulated at HS2 stage. AP2/EREBP transcription factors are part of gene regulatory networks and integrate metabolic, hormonal and environmental signals. The expression level of 34 *AP2-EREBP* transcription factor genes decreased and 7 genes upregulated at HS2 stage (Supplementary Table S8).

### DEGs involved in phenylethanol glycosides biosynthesis

In *C. tubulosa, m*any DEGs involved in PS, PM, and TM pathways were identified between YS1 and HS2 stages (Table 2; Supplementary Table S4). In some gene families, different genes exhibited varied expression levels, and many genes expressed in a very low level. We only selected the genes with relative high expression level (FPKM level over 10) for further analysis.

**Table 2:** Differential expression of genes involved in phenylethanol glycosides biosynthesis

Gene Name	Gene ID	YS1 FPKM	HS2 FPKM	Relative expression level (HS2/YS1)		Reference sequence ID	Expression Trend (HS2/YS1)
				log2 Ratio	Q-value		
*PAL*	isoform_210146	145.88	2.42	-5.97	1.55E-202	XP_011077338.1	down
	isoform_21374	80.55	19.09	-2.14	2.08E-22	XP_011094662.1	down
	isoform_213767	181.18	47.18	-1.96	6.18E-06	XP_011077338.1	down
*C4H*	isoform_15343	3.26	0.18	-3.04	4.37E-02	XP_012838100.1	down
	isoform_189636	1.86	0.51	-1.86	6.77E-03	XP_011089529.1	down
*C3H*	isoform_76310	10.86	0	-8.12	1.64E-13	AYK02617.1	down
	isoform_14095	0.21	21.79	3.59	2.58E-02	AYK02607.1	up
	isoform_10016	329.53	62.13	-2.47	2.07E-36	AYK02617.1	down
	isoform_15416	90.54	13.28	-2.78	1.62E-11	AYK02617.1	down
	isoform_16568	11.08	0.00	-9.16	1.57E-22	AYK02607.1	down
*CSE*	isoform_18401	19.24	2.36	-2.95	2.97E-06	XP_012857987.1	down
	isoform_27751	3.35	28.57	3.02	7.62E-86	XP_012834726.1	up
	isoform_28866	19.68	0.00	-9.48	1.15E-24	XP_012834726.1	down
	isoform_25302	49.96	0.21	-4.35	3.68E-03	XP_011094308.1	down
	isoform_48623	17.34	0.00	-8.60	7.32E-19	XP_012834726.1	down
*CYP98A3*	isoform_12445	12.08	29.56	1.18	5.07E-02	BBB04707.1	up
	isoform_189832	3.71	9.58	1.25	9.76E-03	BBB04707.1	up
*HCT*	isoform_196971	5.12	1.29	-1.99	3.28E-03	PIN19717.1	down
	isoform_31973	24.55	7.77	-1.68	1.04E-03	XP_011094024.1	down
	isoform_122217	27.55	2.90	-2.88	6.93E-03	XP_011094024.1	down
*4CL*	isoform_8492	164.34	13.85	-3.62	3.24E-78	XP_012858335.1	down
	isoform_11132	28.86	15.40	-0.98	1.12E-07	XP_011089239.2	down
	isoform_10866	41.06	11.32	-1.93	1.09E-17	AHL44986.1	down
	isoform_11864	1.54	66.17	5.29	2.27E-63	XP_011099557.1	up
	isoform_118069	11.42	1.00	-3.04	8.73E-03	XP_011084613.1	down
*COMT*	isoform_25614	19.56	0.00	-9.25	2.38E-21	XP_011075886.2	down
	isoform_223053	17.12	2.58	-2.68	6.79E-05	XP_010671879.1	down
	isoform_231223	0.00	23.19	8.56	8.71E-19	Q9XGW0.1	up
	isoform_204033	0.00	30.23	9.66	5.90E-25	XP_011075886.2	up
*CCoAOMT*	isoform_35877	46.86	26.87	-0.87	1.25E-03	XP_012836932.1	down
	isoform_35990	2.16	32.00	3.74	1.11E-16	XP_012836932.1	up
	isoform_230149	2.60	83.89	3.26	3.89E-02	XP_011088234.1	up
*F5H*	isoform_13346	69.06	17.82	-1.97	1.36E-06	XP_011090419.1	down
*PER*	isoform_34903	88.04	19.92	-2.20	4.44E-23	XP_011083218.1	down
	isoform_27857	89.01	10.27	-2.91	4.61E-04	PIN17935.1	down
	isoform_203664	204.81	26.53	-2.92	2.96E-10	PIN15750.1	down
	isoform_25272	9.65	27.98	1.44	1.29E-06	PIN17427.1	up
CCR	isoform_32104	0.00	30.72	9.77	2.00E-26	XP_011090120.1	up
	isoform_91788	0.00	14.99	9.29	2.45E-23	XP_011090120.1	up
*CAD*	isoform_183598	1.18	16.03	3.69	8.09E-04	ADD23217.1	up
	isoform_174357	1.57	193.62	5.50	4.11E-25	ADD23217.1	up
	isoform_107090	1.09	26.79	4.12	1.09E-06	ADD23217.1	up
	isoform_231316	24.34	0.00	-8.72	1.35E-06	ADD23217.1	down
	isoform_228655	135.49	0.35	-8.45	5.73E-19	ADD23217.1	down
*AADC*	isoform_74329	11.29	6.03	-0.98	2.12E-03	PIN14702.1	down
	isoform_15697	300.25	44.24	-2.82	8.99E-66	PIN03085.1	down
*TyDC*	isoform_74329	11.29	6.03	-0.98	2.12E-03	PIN14702.1	down
	isoform_15697	300.25	44.24	-2.82	8.99E-66	PIN03085.1	down
*GOT*	isoform_101670	1.41	4.71	1.66	1.76E-07	XP_012831009.1	up
	isoform_13249	4.54	2.07	-1.19	8.57E-04	XP_011090466.1	down
	isoform_14711	30.70	75.40	1.22	4.16E-35	XP_012847157.1	up
	isoform_17258	57.87	28.08	-1.12	4.77E-12	XP_011090466.1	down
*CuAO*	isoform_75943	11.47	2.24	-2.36	1.56E-05	PIN02817.1	down
	isoform_237680	11.46	5.86	-1.04	5.44E-05	PIN02817.1	down
	isoform_4039	474.68	0.02	-13.49	3.48E-114	PIN02817.1	down
	isoform_4504	90.89	33.39	-1.50	2.40E-04	PIN02817.1	down
*PPO*	isoform_219247	941.05	142.61	-2.79	6.16E-128	XP_011083298.1	down
	isoform_130337	16.14	0.34	-5.51	1.84E-29	PIN00359.1	down
	isoform_10940	265.38	37.65	-2.89	1.67E-137	PIN14145.1	down
	isoform_220219	6.26	18.68	1.50	3.74E-05	XP_011082857.1	up
	isoform_207237	30.46	89.54	1.46	3.57E-05	XP_011082857.1	up
*AADH*	isoform_27810	23.93	2.94	-3.05	2.67E-16	XP_012829622.1	down
	isoform_27930	111.87	49.22	-1.26	3.29E-44	XP_011069785.1	down
	isoform_18708	34.06	74.95	1.06	2.89E-10	PIN17974.1	up
	isoform_158788	1.39	14.55	3.30	6.20E-08	PIM99059.1	up
*UGT*	isoform_18212	19.15	6.30	-1.66	6.95E-14	PIN26543.1	down
	isoform_155690	25.81	5.93	-2.20	2.55E-46	PIN10984.1	down
	isoform_131960	33.88	14.92	-1.25	2.67E-06	PIN15726.1	down
	isoform_215583	63.33	13.82	-2.2	3.27E-06	PIN22174.1	down
	isoform_17110	22.52	136.97	2.44	6.52E-06	PIN07918.1	up

Phenylpropanoid biosynthesis is an important pathway of secondary metabolism in plants. Cinnamic acid, coumaric acid, caffeic acid, and ferulic acid are intermediates of this pathway. These acids can be further converted into coumarin, chlorogenic acid, flavonoids, and lignin. In phenylpropanoid metabolic pathway, most genes families encoding the catalytic enzymes were down-regulated in HS2 stages, including phenylalanine ammonia-lyase, cinnamic acid 4-hydroxylase, and shikimate O-hydroxycinnamoyl-transferase. All four genes encoding Phenylalanine ammonia-lyase (*PAL*), the first key enzyme of PS pathway, were down-regulated in HS2. In some gene families, the expression level of part of the genes were elevated, while other members were decreased, for example, coumarate 3-hydroxylase, 4-coumarate-CoA ligase, caffeoylshikimate esterase, and caffeic acid 3-O-methyltransferase. However, it is worth noting that in these gene families, only one of them has increased expression, while others have decreased expression. For example, there were six transcripts with relative high expression level of *C3H* gene family, among which 4 genes decreased by more than two fold in HS2 to compare with that in YS1, but only one transcript was up-regulated. In *CSE* gene family, there were nine transcripts, among which the expression of four genes were greatly reduced while one transcript was increased. Three genes showed elevated expression in HS2, including 5-O-(4-coumaroyl)-D-quinate 3’-monooxygenase, caffeoyl-CoA O-methyltransferase, and Cinnamoyl-CoA reductase genes. CYP98A3 functions to catalyze p-Coumaroyl quinic acid to caffeoyl quinic acid, or p-Coumaroyl shikimic acid to caffeoyl shikimic acid, respectively. Four *CYP98A3* genes were found in *C. tubulosa*, two of them had elevated expression in HS2 stage. CCR and CCoAOMT are key enzymes of PS pathway and related to lignin synthesis. Two *CCR* genes were expressed highly at HS2, while almost undetectable at YS1. The expression changes of these genes suggested that the synthesis of phenylethanoside precursors, such as caffeic acid and ferulic acid, were channeled to lignin synthesis in HS2.

TM pathway was the main pathway for phenylethanol aglycon synthesis in *Rehmannia glutinosa* [35]. And PM pathway was speculated the pathway for synthesizing phenylethanol aglycon in *C. deserticola* [17]. Aromatic-L-amino-acid decarboxylase (*AADC*), copper amine oxidase (*CuAO*), and aryl-alcohol dehydrogenase are present in both TM and PM pathways. We found two *AADC* genes and seven *CuAO* genes were all down-regulated in HS2. In *Rehmannia glutinosa* and *Petroselinum crispum*, tyrosine/DOPA decarboxylase catalyzes (*TyDC*) decarboxylation of tyrosine to Tyramine [35, 36]. In *C. tubulosa*, the expression levels of five members of tyrosine/DOPA family were all very low, and there was no significant difference between two stages. Therefore, we suggested that *AADC* plays an important role in this decarboxylation process in *C. tubulosa,* rather than *TyDC.* The similar situation may happen with the deamination of tyrosine, because the tyrosine aminotransferase and histidinol-phosphate aminotransferase expressed lowly and unchanged between two stages.

Glycosylation is one of the final steps involved in the biosynthesis of many plant secondary metabolites. UDP-glycosyltransferase (UGT) can transfer sugar moieties from active sugar molecules (e.g. UDP-glucose) to various acceptor molecules. There were 28 *UGT* genes identified highly expressed in this study, and 13 of which were down-regulated and three were up-regulated in HS2. The shikimate O-hydroxycinnamoyltransferase was inferred involved in catalyzing the acyl-transfer from coenzyme A-activated acids to phenylethanol aglycon [36]. We identified 13 unigenes encoding shikimate O-hydroxycinnamoyltransferase, and two of which were down-regulated in HS2.

## Discussion

The dried succulent stems of *Cistanche* were widely used in traditional Chinese medicines. *C. deserticola* and *C. tubulosa* are the two main medicinal plants [37]. The genomic and transcriptomic resources of *Cistanche* are mainly from *C. deserticola* [15–17]. In the current study, we analyzing the stem full-length transcriptome of *C. tubulosa* using PacBio SMRT Sequel platform, and obtained 237,772 unique transcripts. The proportion of the annotated transcripts using the seven software and several public databases is only 79.12%, while in the case of general plants, the proportion is about 90% [23].

Due to the biological activity of PhGs, the biosynthetic pathway has been extensively studied in order to obtain PhGs rich medicinal materials. The putative PhGs biosynthesis pathway was established based on precursor feeding experiments. Feeding tyrosine and phenylalanine to cell suspension culture of *Cistanche* can increase the accumulation of acteoside, echinoside or 2’-acetyl acteoside [38, 39]. Isotope labeled feeding studies in *Syringa vulgaris* and *Olea europaea* showed that tyrosine to 3,4-dihydroxytyrosol (DHPA) conversion was through dopamine or tyramine pathway, and the conversion ofphenylalanine to coffeyl moiety was through phenylpropane pathway [40, 41]. It recognized that the organic acid acyl moiety (coffeyl, feruloyl or coumaroyo) of PhGs were generated from phenylpropanoid synthesis pathway, while the synthesis of phenylethanol moiety has several possible choices. In *Rehmannia glutinosa*, phenylethanol part was considered to be started from the tyrosine precursors by the tyrosine-derived pathway [35, 42, 43]. In *C. deserticola*, the synthesis of phenylethanol part is reported presumably from two pathways. One is the caffeic acid or ferulic acid pathway, which is part of the PS pathway. The other is the PM pathway, in which the phenylethanol was converted to phenylethanol aglycon [17].

**Fig. 6:**
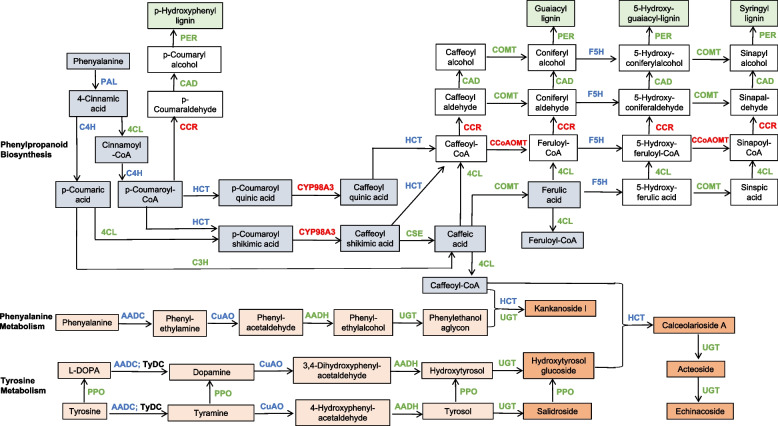
The proposed pathways and genes involved in the biosynthesis of PhGs and lignins in *C. tubulosa*.  The grey box represents the organic acid acyl moiety of PhGs derived from PS pathway;  The orange box represents the phenylethanol moiety or phenylethanol glycosides that derived from TM/PM pathways;  The pink box represents metabolites in tyrosine/phenylalanine metabolism pathways;  The green box represents lignins derived from the PS pathway

Studies indicated that tyrosine could be converted to 4-hydroxyphenyl- acetaldehyde by aminotransferase and 4-hydroxyphenylacetaldehyde synthase though 4-hydroxyphenylpyruvate [35, 44]. In this pathway, tyrosine aminotransferase (*TAT*)*,* histidinol-phosphate aminotransferase (*hisC*), and aspartate aminotransferase (*GOT*) are the main active enzymes. We found that both *TAT* and *hisC* genes expressed lowly and unchanged in YS1 and HS2. In *GOT* gene family, one gene was up-regulated and one gene was down-regulated in HS2, the changing trend of expression was not obvious. Meanwhile, we didn’t found 4-hydroxyphenylpyruvate decarboxylase (*4HDPC*) coding gene in the full-length transcripts of *C. tubulosa*. Therefore, we suggested that the 4-hydroxyphenylpyruvate pathway maybe not the primary one in *C. tubulosa*. Based on these results, we proposed a potential model of PhGs biosynthesis regulation in *C. tubulosa* **(Fig. ****6****)**. In summary, the phenylethanol part is derived from two parallel pathways, the PM pathway and TM pathway in *C. tubulosa*. In the PM pathway, phenylalanine is decarboxylated under the action of aromatic-L-amino-acid decarboxylase to produce phenylethylamine, and then generate phenylethylalcohol under copper amine oxidase and aryl-alcohol dehydrogenase. In the TM pathway, tyrosine or its oxidation product L-DOPA were first decarboxylated to produce tyramine or dopamine, then to tyrosol or hydroxytyrosol under the action of CuAO and AADH, respectively. However, the acyltransferase corresponding gene is still uncertain, which transfers caffeoyl- group to phenylethanol aglycon in the downstream of PhGs synthesis. Inferred from transcriptome data, shikimate O-hydroxycinnamoyltransferase may play the role, but the specific catalytic function requires further study.

There were significant differences in gene expression between YS1 and HS2 stages in *C. tubulosa*, and the expression level of many catalyzing enzyme genes related to PhGs synthesis were decreased. The expression level of catalyzing enzyme genes that control the flow to lignin were increased in HS2 (*CCR* and *CCoAOMT*) (Fig. 6). The result indicated that the synthesis of PhGs was weakened while the synthesis of lignin was activated at HS2 stage, which supported the rationality of harvest the succulent stem of *C. tubulosa* at this stage in practice. The regulators of PhGs biosynthetic pathway are still uncertain, however, some transcription factors have been found to regulate the expression of key enzyme genes in phenylpropanoid pathway [30–32, 45, 46]. In *Arabidopsis*, overexpression of the MYB transcription factor resulted in activation of genes across the entire phenylpropanoid pathway, including genes such as *AtPAL1* and *At4CL* [30–32]. In *Plagiochasma appendiculatum*, the overexpression of bHLH transcription factor also upregulated *PaPAL* and *Pa4CL1* [45]. In *WRKY1* transgenic tomato, researchers found that transcript of key genes from phenylpropanoid pathway accumulated [46]. Our results showed that the expression of *WARK*, *MYB*, and *bHLH* transcription factors changed significantly, the dramatic gene expression changes in these transcription factors suggested their potential regulatory functions in PhGs synthesis pathway.

## Conclusions

In the present study, the full-length transcriptome and gene expression profiling of *C. tubulosa* stem in different developmental stages were achieved. Key genes of catalyzing enzymes for PhGs biosynthesis were identified, and a model for PhGs biosynthesis of *C. tubulosa* was proposed. The expression patterns of key genes were consistent with the accumulation of PhGs. These results laid the foundation for further studies on molecular mechanism of PhGs biosynthesis and regulation in *C. tubulosa*.

## Supplementary Information

**Additional file 1: Supplementary Figure S1.** BUSCO assembly evaluation results. C (complete): matches the BUSCO database sequence; F (fragmented): only part of the sequence can be compared with the BUSCO database; D (duplicate): multiple genes are compared with the same BUSCO; M (missing): the filtered sequence.

**Additional file 2: Supplementary Figure S2.** Transcription factor family classification. The X-axis represents the corresponding number of isoforms, the Y-axis represents the transcription factor family classification.

**Additional file 3: Supplementary Table S1.** Seven databases showed the annotation results of the full-length transcripts*.*

**Additional file 4:** **Supplementary Table S2. **Coding ability prediction results of the full-length transcripts*.*

**Additional file 5:** **Supplementary Table S3.** Transcription factor families and transcription factor genes.

**Additional file 6: Supplementary Table S4.** Differential expression of genes involved in PS, PM and TM pathways.

**Additional file 7: Supplementary Table S5.** Differentially expressed genes between HS2 and YS1.

**Additional file 8: Supplementary Table S6.** KEGG pathways of DEGs between HS2 and YS1.

**Additional file 9: Supplementary Table S7.** Primers for qRT-PCR analysis.

**Additional file 10: Supplementary Table S8.** Differential expression of transcription factors genes.

## Data Availability

Raw sequence reads of the RNA-seq data were available in NCBI Short Read Archive (SRA) Database (Bioproject: PRJNA786765). The assembled full length transcriptome data have been deposited in NCBI Transcriptome Shotgun Assembly (TSA) Database (TSA submission: SUB10777875). **Ethical approval and consent to participate.** The *Cistanche tubulosa* used in this study was cultivated. The materials were collected from plants grown in the experimental station of Shandong Academy of Agricultural Sciences.

## Abbreviations

PhGs: Phenylethanol glycosides
PS: Phenylpropanoid synthesis pathway
TM: Tyrosine metabolism pathway
PM: Phenylalanine metabolism pathway
PhGs: Phenylethanol glycosides
PAL: Phenylalanine ammonia-lyase
C4H: Cinnamic acid 4-hydroxylase
C3H: Coumarate 3-hydroxylase
CSE: Caffeoylshikimate esterase
CYP98A3: 5-O-(4-coumaroyl)-D-quinate 3’-monooxygenase
HCT: Shikimate O-hydroxycinnamoyltransferase
4CL: 4-Coumarate-CoA ligase
COMT: Caffeic acid 3-O-methyltransferase
CCoAOMT: Caffeoyl-CoA O-methyltransferase
F5H: Ferulate-5-hydroxylase
PER: Peroxidase
CCR: Cinnamoyl-CoA reductase
CAD: Cinnamyl alcohol dehydrogenase
AADC: Aromatic-L-amino-acid decarboxylase
TyDC: Tyrosine/DOPA decarboxylase
GOT: Aspartate aminotransferase
hisC: Histidinol-phosphate aminotransferase
TAT: Tyrosine aminotransferase
PPO: Copper amine oxidase (CuAO) Polyphenol oxidase
AADH: Aryl-alcohol dehydrogenase
UGT: UDP-glycosyltransferase
4HDPC: 4-Hydroxyphenylpyruvate decarboxylase
DEGs: Differentially expressed genes
YS1: Young fleshy stem
HS2: Harvest stage
FPKM: Fragments Per Kilobase of transcript per Million fragments
