# Supplementary figures and images for: De novo full length transcriptome analysis and gene expression profiling to identify genes involved in phenylethanol glycosides biosynthesis in Cistanche tubulosa
Source: BMC Genomics. 2022 Oct 8;23:698. doi: 10.1186/s12864-022-08921-x (PMC9548140; doi:10.1186/s12864-022-08921-x)

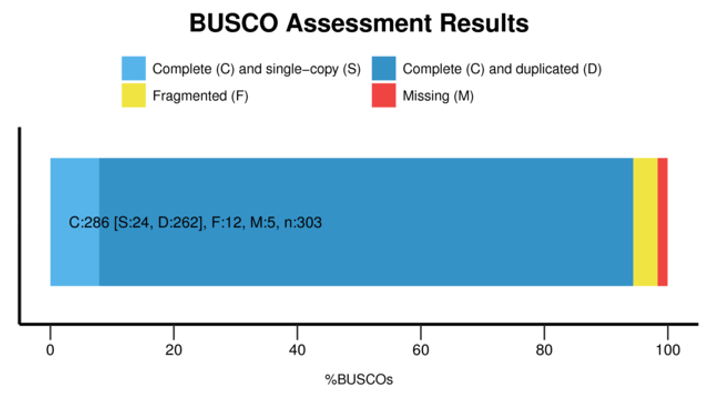

Supplement: Supplementary file 1 — Additional file 1: Supplementary Figure S1. BUSCO assembly evaluation results. C (complete): matches the BUSCO database sequence; F (fragmented): only part of the sequence can be compared with the BUSCO database; D (duplicate): multiple genes are compared with the same BUSCO; M (missing): the filtered sequence. [file 12864_2022_8921_MOESM1_ESM.png]

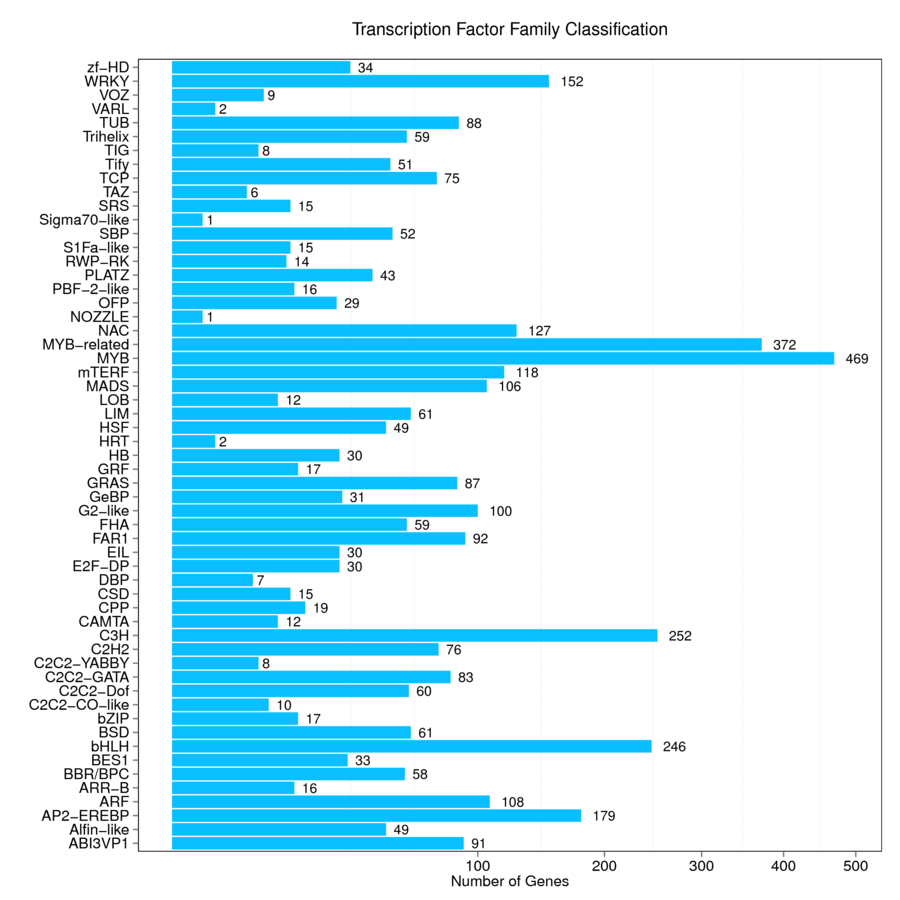

Supplement: Supplementary file 2 — Additional file 2: Supplementary Figure S2. Transcription factor family classification. The X-axis represents the corresponding number of isoforms, the Y-axis represents the transcription factor family classification. [file 12864_2022_8921_MOESM2_ESM.png]
